# Application of PMCA to screen for prion infection in a human cell line used to produce biological therapeutics

**DOI:** 10.1038/s41598-019-41055-x

**Published:** 2019-03-19

**Authors:** Adam Lyon, Charles E. Mays, Frank Borriello, Glenn C. Telling, Claudio Soto, Sandra Pritzkow

**Affiliations:** 1 Mitchell Center for Alzheimer’s Disease and Related Brain Disorders, University of Texas McGovern Medical School, Houston, TX 77030 USA; 2 Alloplex Biotherapeutics, Inc., 21 Erie Street, Cambridge, MA 02139 USA; 3 Prion Research Center, Colorado State University, Colorado, USA

## Abstract

Advances in biotechnology have led to the development of a number of biological therapies for the treatment of diverse human diseases. Since these products may contain or are made using human or animal (e.g. cattle) derived materials, it is crucial to test their safety by ensuring the absence of infectious agents; specifically prions, which are highly resilient to elimination and produce fatal diseases in humans. Many cases of iatrogenic Creutzfeldt-Jakob disease have been caused by the use of biological materials (e.g. human growth hormone) contaminated with prions. For this reason, it is important to screen cells and biological materials for the presence of prions. Here we show the utility of the Protein Misfolding Cyclic Amplification (PMCA) technology as a screening tool for the presence of human (vCJD) and bovine (BSE) prions in a human cell therapy product candidate. First, we demonstrated the sensitivity of PMCA to detect a single cell infected with prions. For these experiments, we used RKM7 cells chronically infected with murine RML prions. Serial dilutions of an infected cell culture showed that PMCA enabled prion amplification from a sample comprised of only one cell. Next, we determined that PMCA performance was robust and uncompromised by the spiking of large quantities of uninfected cells into the reaction. Finally, to demonstrate the practical application of this technology, we analyzed a human cell line being developed for therapeutic use and found it to be PMCA-negative for vCJD and BSE prions. Our findings demonstrate that the PMCA technology has unparalleled sensitivity and specificity for the detection of prions, making it an ideal quality control procedure in the production of biological therapeutics.

## Introduction

Biological therapeutic products derived from living organisms are emerging as a viable method to prevent and treat a variety of human diseases. Many biological treatments involve the transmission of material between people. Globally, about 85 million blood transfusions and 125,000 organ transplants are performed each year and continue to rise^1^. New scientific discoveries continue to expand the field of biological therapeutic products, thus increasing the frequency of patient exposure. For instance, the rapidly growing stem cell field is poised to contribute to a large increase in these types of interventions. New technological and scientific discoveries have expanded the range of indications for stem cell treatment to include diseases of the brain, like traumatic brain injury^2^, stroke^3^, Alzheimer’s^4^, Huntington’s^5^ and Parkinson’s^6^.

The growing use of biological therapeutic products makes it increasingly crucial to ensure patient safety, specifically in regards to reducing the risk of disease transmission from donor to recipient. Earlier cases of transmission including human immunodeficiency virus (HIV) and hepatitis C virus (HCV) through blood transfusions have guided the implementation of procedures to reduce these risks^7^. These include medical history reviews of donors and sample testing when possible. Immunoassays and nucleic acid amplification assays are used to detect viruses, bacteria, and other micro-organisms, which can be transmitted during blood transfusions, vaccination, tissue and organ transplantations, and other cellular therapies. Prions also have the potential to be transmissible during these interventions, but are undetectable by conventional methods^7^. Reducing the risk of transmission relies solely on reviewing the donor’s medical history and excluding people who have lived in areas of high exposure to prion infection.

Prions are responsible for a group of fatal neurodegenerative diseases affecting humans and various mammalian species^8^. The sole component of the infectious agent is a misfolded form (PrP^Sc^) of the host-encoded prion protein. When PrP is folded into its natural, non-infectious conformation, it is denoted PrP^C^. The disease is caused by the accumulation of PrP^Sc^, which is created by a self-templated conversion of PrP^C^ to PrP^Sc^ (refs^9,10^). The initial PrP^Sc^ seeds can develop spontaneously as in the case of sporadic Creutzfeldt Jakob Disease (sCJD). Several rare, hereditary mutations in the gene encoding PrP increase the likelihood of PrP^Sc^ formation. Distinct mutations cause different diseases including Gerstmann–Sträussler–Scheinker syndrome, familial CJD, and fatal familial insomnia^11^. Alternatively, PrP^Sc^ can be transmitted through contaminated materials. For example, a variant CJD (vCJD) in humans is caused by the consumption of beef from cattle infected with bovine spongiform encephalopathy (BSE) prions^12^. Human to human transmission has also occurred in medical procedures causing iatrogenic CJD (iCJD)^13^.

There are many challenges to limit prion transmission. First, PrP^Sc^ is highly resistant to common disinfection techniques^14^. Second, prion diseases are characterized by a long incubation period during which PrP^Sc^ accumulates in the brain and peripheral tissues while not producing symptoms^15^. During this period, it is possible for an individual to make a blood or tissue donation ignorant to his or her own infection. Therein lies the final challenge: detecting prions during the clinically-silent, incubation period^16^. The standards for such detection assays should be at least 99.5% specificity and sensitivity as recommended by the World Health Organization^17^. Only until recently has this been achieved for prion detection. In 2001, the Protein Misfolding Cyclic Amplification (PMCA) technique was first used to amplify PrP^Sc^ *in vitro* for biochemical detection of prions^18^. Automation and other improvements enabled highly efficient prion detection by PMCA, which was recently shown to detect prions in blood of vCJD patients with 100% sensitivity and 100% specificity^19,20^. A similar level of sensitivity was obtained in urine samples^21^.

In this study, we demonstrate the application of PMCA for detection of prions in a human cell line under development to produce vaccines for human use. Specifically, we report that the technology has the potential to detect a single prion-infected cell, and we provide an example of its use to screen an allogeneic whole cell therapeutic vaccine candidate derived from a human tumor cell line.

## Materials and Methods

### Cells

The cellular therapeutic candidate being developed by Alloplex Biotherapeutics derives from the SK-MEL2 line, originally established in the 1970’s and currently maintained by the NIH. These cells were provided to Alloplex by the DCTD Tumor Repository, National Cancer Institute at Frederick, Maryland.

RK13 rabbit kidney cells stably expressing mouse PrP^C^, called RKM7, were used in these experiments. RKM7 cells chronically infected with murine adapted scrapie prions (RML) are denoted RKM7-RML. Cultures of RKM7, RKM7-RML and Alloplex cells were counted, pelleted and resuspended in PBS. For the detection of prions from infected cells, RKM7-RML cells were serially diluted in PBS to achieve 10^5^,10^4^, 10^3^, 100, 10, and 1 cell per 200 µL.

### Sample Processing and PMCA procedure

All cell materials were processed prior to PMCA as described previously^19^. Briefly, after one hour of incubation in 10% sarkosyl, samples underwent two rounds of ultracentrifugation separated by a PBS wash. Pelleted samples were resuspended in 100 µL of the appropriate PMCA substrate and transferred to 0.2 mL PCR tubes. Pellets from RKM7-RML cells were resuspended directly into wild type (WT) mouse 10% brain homogenate. The Alloplex cell pellet (approximately 3 × 10^6^ cells) was resuspended in 3.6 mL of PBS and divided into sixteen 200 µL aliquots containing 166,667 cells. Four aliquots were used for detection of human prions, and 4 for detection of bovine prions. From the remaining aliquots, two aliquots each were for spiked with 10^−6^ and 10^−8^ dilutions of vCJD and BSE brain homogenates. The PBS cell suspensions were processed by sarkosyl extraction, ultracentrifugation, and resuspended in the appropriate PMCA substrate. As PMCA substrate, 10% brain homogenates from transgenic mice expressing human (TgHu) and bovine (TgBov) PrP^c^ at levels 16- and 4- folds higher than the endogenous protein, were used for amplification of human and bovine prions, respectively. TgHu and TgBov substrates were prepared in conversion buffer (PBS supplemented with 150 mM NaCl and 1% Triton X-100) with EDTA-free protease inhibitor and supplemented prior to PMCA with 6 mM EDTA, 100 µg/mL Heparin, and 0.05% Digitonin. WT mouse substrate was prepared in conversion buffer with protease inhibitor containing EDTA (5 mM).

PMCA was carried out as described previously^22^. Briefly, samples were incubated at 37 °C for 29 minutes and 30 seconds followed by 30 seconds of sonication at an amplitude of 25 using a Qsonica sonicator.

### Western Blot

PMCA products were digested by proteinase K (PK; 100 µg/mL) for 1 hour in a thermomixer set at 600 rpm and 37 °C. Digestion was terminated by the addition of sample buffer and incubation for 10 minutes at 100 °C. After SDS-page and transfer to a nitrocellulose membrane, protease resistant PrP was probed with the 6D11 antibody (1:30000 dilution).

## Results

**Figure 1:**
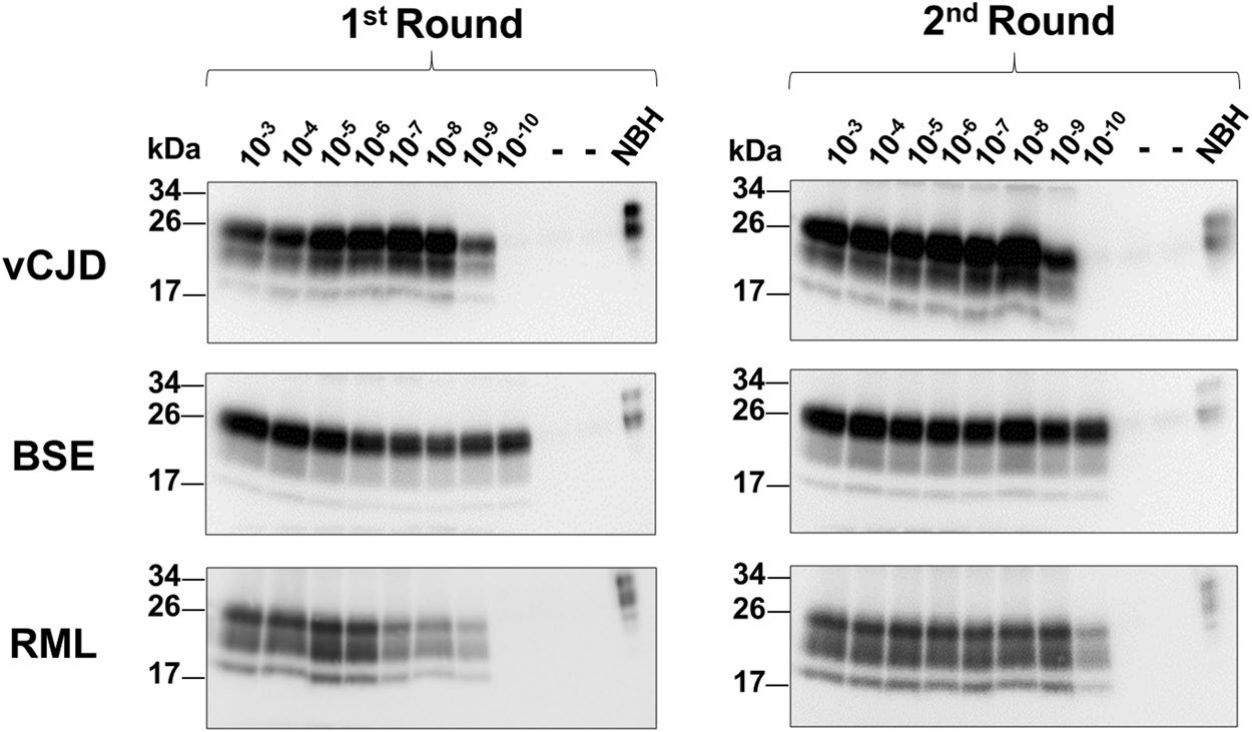
PMCA amplification efficiency. Serial dilutions (10^−3^ to 10^−10^) of vCJD, BSE, and RML prions were made in 10% brain homogenates as substrate (TgHu, TgBov, WT). PMCA products from the first (left) and second rounds (right) were PK digested (100 µg/mL) and analyzed by western blot. PrP was probed using the 6D11 antibody. Unspiked brain homogenate was used as negative PMCA control (−). NBH not treated with PK was used as migration controls. Numbers on the left indicate the position of molecular weight markers.

The goal of this study was to detect prion contamination in a cell line used to produce therapeutic products intended for human use. For this reason, we focused on prions that have been shown to be infectious to humans, i.e. prions associated with vCJD and BSE. To demonstrate the ability of PMCA for ultrasensitive detection of prions from these sources, we first performed experiments to analyze the efficiency of the assay and its limit of detection. For this purpose, we made serial dilutions of vCJD and BSE prions from infected brains in PMCA substrate prepared from TgHu and TgBov mice, respectively. As a control, we also used murine RML prions amplified with WT mouse brain homogenate. Infected brain seeds were prepared as 10% homogenate (10^−1^) and serially diluted from 10^−3^ to 10^−10^ before subjecting to 2 rounds of PMCA. After a single round of PMCA, RML and vCJD prions were detectable up to the 10^−9^ dilution (Fig. 1), while BSE was detectable up to the 10^−10^ dilution. After an additional round of PMCA, the 10^−10^ dilution of RML prions was detected (Fig. 1). Differences in the maximum dilution of brain homogenate detectable by PMCA do not necessarily reflect variability on the amplification efficiency, but rather distinct amounts of PrP^Sc^ in the piece of the brain used as inoculum. In our experience, the maximum dilution detectable by PMCA ranges between 10^−9^ and 10^−11^ and likely corresponds to the dilution containing single particles of PrP^Sc^ seeds^23,24^.

**Figure 2:**
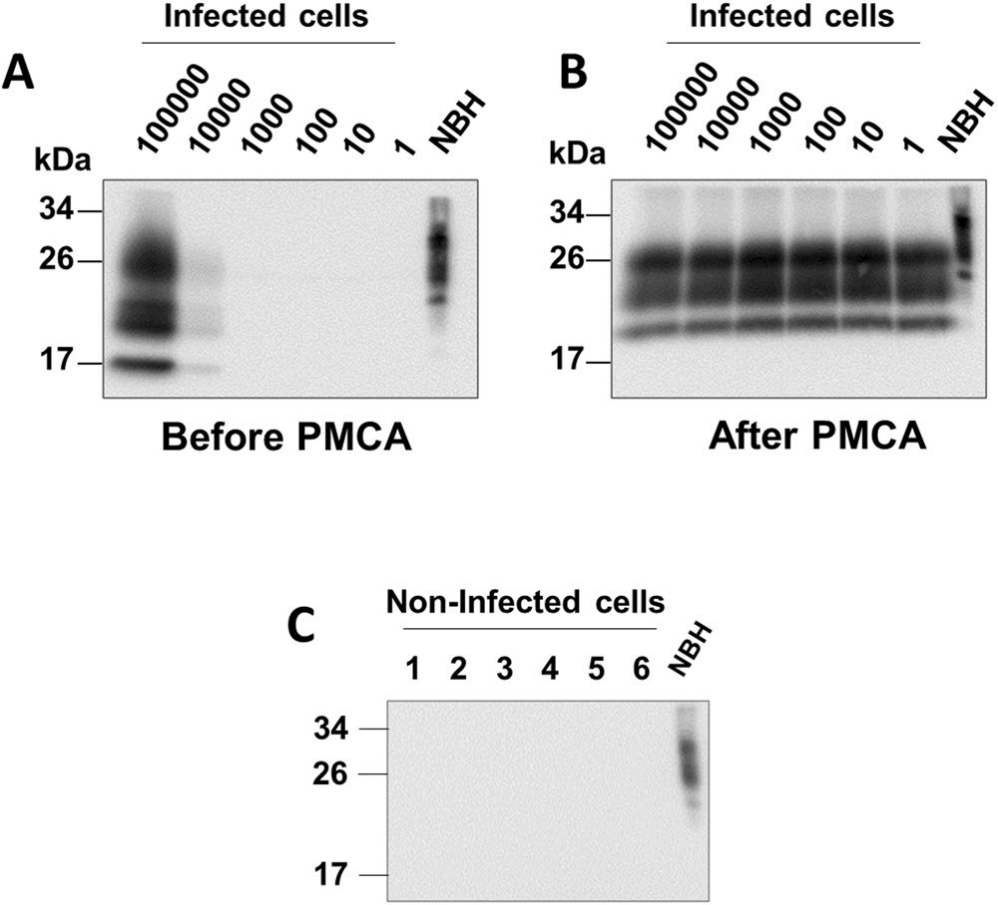
PrP^Sc^ detection from different quantities of RML-infected cells. Western blots of RKM7-RML cell dilutions before (**A**) and after (**B**) three rounds of PMCA amplification. Counted cells were thoroughly resuspended in PBS and serial dilutions were made to achieve 100000, 10000, 1000, 100, 10, and 1 cells. Panel C shows 6 replicates of RKM7 cells not infected with prions. Samples were PK treated (100 µg/mL) and subjected to western blot using the 6D11 anti-PrP antibody. NBH from WT mouse (without PK treatment) was used as a migration control. Numbers on the left of each blot indicate the position of molecular weight markers.

Since the goal of this study was to detect the putative presence of prion-infected cells in a culture intended for human therapeutic use, we next analyzed whether PMCA can detect prions attached to cells in culture and specifically whether the assay can detect a single infected cell. To do this, we used, as a model, a culture of cells chronically infected with RML prions (RKM7-RML). This cellular model was previously established and has been shown to support prion propagation indefinitely in culture^25^. Cells were counted and serially diluted in PBS to achieve aliquots containing on average 100000, 10000, 1000, 100, 10 and 1 cells. By western blot without PMCA, a strong signal for PrP^Sc^ was seen for 100000 cells (Fig. 2A). A faint signal can be seen from 10000 cells. No signal is detectable in lower amount of cells without amplification. After three rounds of PMCA, we detected PrP^Sc^ from all samples, even those containing an estimated single cell (Fig. 2B). No signal was detected in non-infected cells (Fig. 2C). This data indicates that PMCA has the power to identify 1 infected cell in a pool of millions of non-infected cells. However, it is important to highlight that these experiments were done with cells chronically infected with RML mouse prions. Considering that the efficiency of PMCA to amplify RML, vCJD and BSE prions is comparable (Fig. 1), we believe it is reasonable to assume that the results with RML will be reproduced with cells infected with human or bovine prions. Unfortunately, despite extensive efforts from diverse investigators, it has not been possible to generate cells consistently infected with human prions.

**Figure 3:**
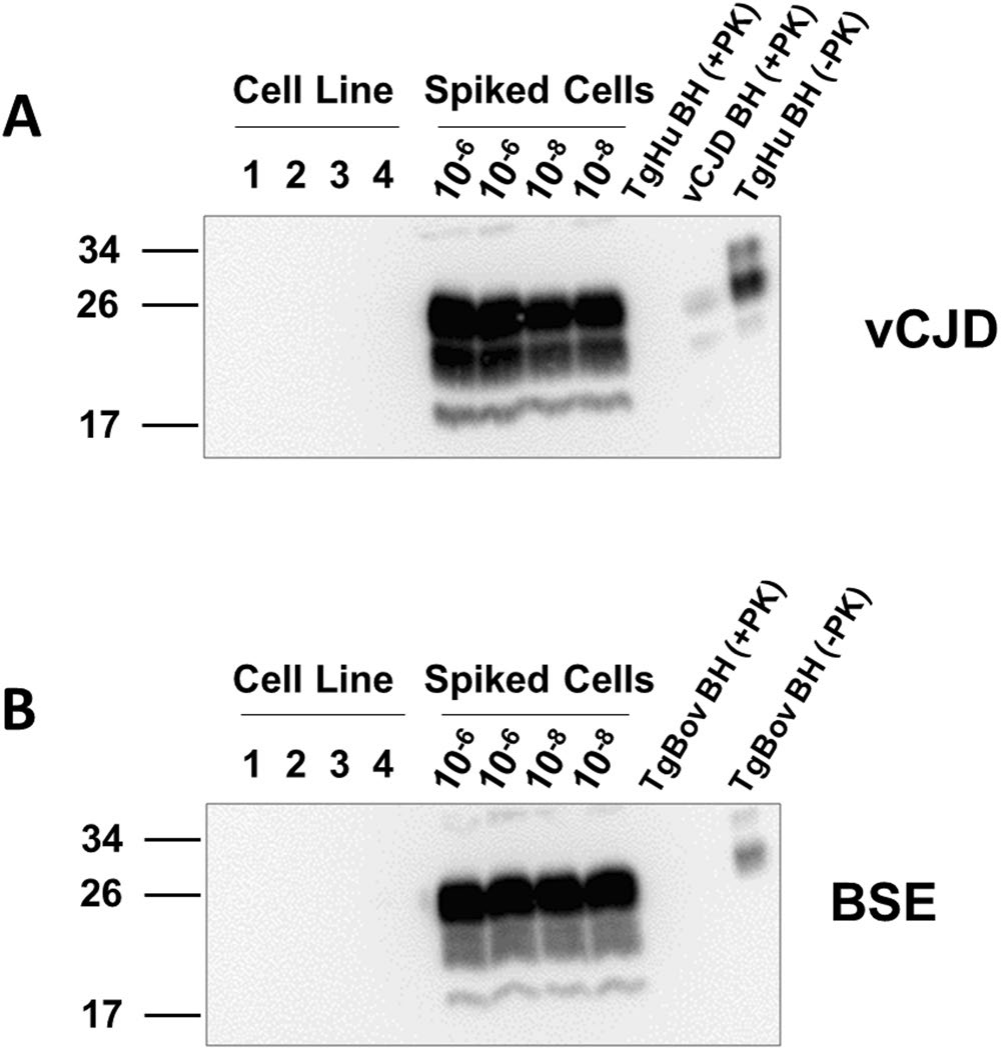
Screening for prion infection in cells used for production of biological therapeutics. A pellet of 3 × 10^6^ cells from the NIH SK-MEL2 line was resuspended in 3.6 mL of PBS and 200 μl aliquots were tested for the presence of vCJD and BSE prions in 4 replicates. Cells were processed as described in Methods. Pellets were resuspended in 100 µL of TgHu or TgBov 10% brain homogenate and transferred to PMCA tubes. As a positive control, aliquots of SK-MEL2 cell pellets were spiked with 10^−6^ or 10^−8^ dilutions of vCJD or BSE brain homogenates. Controls were processed alongside the experimental unspiked samples. The figure shows the Western blot results from the third round of PMCA following PK (100 µg/mL) treatment for the experiment aiming to detect human (**A**) and bovine (**B**) prions. TgHu or TgBov brain homogenate (BH) with or without PK digestion was used as a migration control. 6D11 antibody was used to probe PrP. Numbers on the left of each blot indicate the position of molecular weight markers.

With the system validated to detect prions in an estimated single infected cell, we proceeded to test a cell line used to produce biological products. Specifically, we tested a cell line under development by Alloplex Biotherapeutics to produce vaccine candidates for cancer therapy, under development by Alloplex Biotherapeutics, Inc. A pellet of an estimated 3 × 10^6^ cells was resuspended in PBS, divided in aliquots, and processed by sarkosyl extraction and ultracentrifugation. After removal of the supernatant, processed pellets were resuspended in 10% brain homogenate of transgenic mice expressing the human or bovine prion protein. Samples were run in four replicates along with positive controls corresponding to a PMCA tube containing the same amount of Alloplex cells spiked with 10^−6^ and 10^−8^ dilutions of vCJD or BSE. The results with the Alloplex cells were clearly negative in all PMCA rounds and all replicates (Fig. 3). All positive controls gave the expected results (Fig. 3), indicating the system was performing at its highest efficiency and reproducibility.

## Discussion

As of 2017, around 492 cases of human prion disease have been transmitted iatrogenically by a variety of medical procedures including organ transplants, tissue grafts, blood transfusions, and the use of human-derived therapeutic products (e.g. growth hormone)^26^. The donor tissues were not suspected to contain prions, and the contamination was only considered once the recipient developed disease. A key strategy to reducing risk of iatrogenic transmission of prions would depend on the availability of a technology that can be used to screen for prion infection in biological samples aimed for human use. Given that prion infection can be achieved even with a very small quantity of infectious prions (equivalent to a ~10^−8^ dilution of infected brain homogenate), the methodology needs to have an extremely high sensitivity and specificity. In recent years, we and others have shown that amplification technologies, such as PMCA and real-time quaking induced conversion (RT-QuIC), enable ultrasensitive detection of prions from a variety of samples^22,27^. These technologies employ the prion principle to amplify the amount of PrP^Sc^ present in a sample. We and others have shown that PMCA efficiently detects prions associated with vCJD in samples of human blood and urine with sensitivities and specificities approaching 100%^19–21^. The technique was also shown to detect vCJD PrP^Sc^ in blood during the pre-symptomatic stage of the disease^28^. We are currently validating the PMCA technology for commercial use in screening of blood banks for potentially contaminated samples, thus preventing new cases. This is highly relevant considering that contamination of blood products has been a major concern following the BSE outbreak in the UK. It has been estimated that 1 in 2000 people are currently subclinical carriers of vCJD prions^29^. Indeed, blood transfusion has been linked to the iatrogenic transmission of vCJD^30–32^.

The majority of iatrogenic prion cases have been caused by dura mater grafts and human growth hormone treatments, but a potential risk of other tissues and biological samples remains^26^. For example, PMCA analysis of various tissues from 4 vCJD patients revealed a wide distribution of infectious prions in peripheral tissues, including kidney, lung, and bone marrow among many others that are relevant for organ transplantation^33^. It is important to mention that vCJD and iCJD account for 2–5% of CJD cases. sCJD and gCJD account for 85–90% and 10% respectively^34^. We are working to adapt PMCA to amplify and detect sCJD and gCJD prions, which will allow for a better understanding of the peripheral distribution of PrP^Sc^ in these diseases and the associated risks of iatrogenic transmission.

A rapidly increasing field of biological therapeutics involves the use of cellular products (e.g. recombinant proteins and vaccines) and stem cells for regenerative treatment. In this study, we explored the application of PMCA to screen cells utilized for the production of biological therapeutics. Our data demonstrate that PMCA enabled detection of PrP^Sc^ from an estimated single infected cell. Compared to the results of detection by western blot, PMCA exhibits a >10^5^ higher sensitivity. These findings demonstrate the extreme power of PMCA, which can detect one infected cell in a culture of one million or more non-infected cells. It is difficult to rule out that some PrP^Sc^ may have been present in the medium and carried over during the dilutions; however, in practical application this means that the aliquot of the sample may not need to have captured a single infected cell to result in a positive signal. As an example of the application of the PMCA technology to screen biological therapeutics under development for human use, we tested a cell line that is being used to produce anti-cancer vaccines. Our data clearly shows that this sample was free of vCJD and BSE prions. Further studies are needed to expand the application of the technique to test more cell lines and tissues for transplantation.

Future research may also attempt to optimize the PMCA assay to screen biological products for other misfolded protein aggregates implicated in more prevalent neurodegenerative diseases, such as amyloid-beta (Aβ) and tau in Alzheimer’s disease and alpha-synuclein (α-syn) in Parkinson’s disease. Indeed, recent evidence in cellular and animal models of these diseases suggests that Aβ, tau and α-syn misfolded protein aggregates may also spread by the prion principle^35,36^, although the extent to which this process occurs in humans is yet unknown. Importantly, we and others have shown that the PMCA and RT-QuIC amplification technologies can be successfully adapted for high sensitive detection of Aβ, tau and α-syn aggregates in human biological fluids^37–41^, offering the promise that this assay could be used for biochemical diagnosis of neurodegenerative diseases and perhaps to further increase the safety of biological products.

## References

[1] Carson JL (2012). Red blood cell transfusion: a clinical practice guideline from the AABB*. Ann. Intern. Med..

[2] Cox CS (2017). Treatment of Severe Adult Traumatic Brain Injury Using Bone Marrow Mononuclear Cells. Stem Cells.

[3] Steinberg GK (2016). Clinical Outcomes of Transplanted Modified Bone Marrow–Derived Mesenchymal Stem Cells in Stroke. Stroke.

[4] Bali P, Lahiri DK, Banik A, Nehru B, Anand A (2017). Potential for Stem Cells Therapy in Alzheimer’s Disease: Do Neurotrophic Factors Play Critical Role?. Curr. Alzheimer Res..

[5] Haddad MS, Wenceslau CV, Pompeia C, Kerkis I (2016). Cell-based technologies for Huntington’s disease. Dement. Neuropsychol..

[6] Yasuhara T, Kameda M, Sasaki T, Tajiri N, Date I (2017). Cell Therapy for Parkinson’s Disease. Cell Transplant..

[7] Di Minno G (2016). Current concepts in the prevention of pathogen transmission via blood/plasma-derived products for bleeding disorders. Blood Rev..

[8] Aguzzi A, Calella AM (2009). Prions: protein aggregation and infectious diseases. Physiol. Rev..

[9] Soto C (2011). Prion hypothesis: the end of the controversy?. Trends Biochem. Sci..

[10] Prusiner SB (1998). Prions. Proc. Natl. Acad. Sci. USA.

[11] Ironside JW, Ritchie DL, Head MW (2018). Prion diseases. In Handbook of clinical neurology.

[12] Collinge J (1999). Variant Creutzfeldt-Jakob disease. Lancet (London, England).

[13] Hamaguchi T (2009). The risk of iatrogenic Creutzfeldt-Jakob disease through medical and surgical procedures. Neuropathology.

[14] Taylor DM (2000). Inactivation of Transmissible Degenerative Encephalopathy Agents: A Review. Vet. J..

[15] Hill AF, Collinge J (2003). Subclinical prion infection in humans and animals. Br. Med. Bull..

[16] Soto C (2004). Diagnosing prion diseases: needs, challenges and hopes. Nat. Rev. Microbiol..

[17] World Health Organization. Screening Donated Blood for Transfussion-Transmissible Infections: Recomendation (2009). 23741773

[18] Saborio GP, Permanne B, Soto C (2001). Sensitive detection of pathological prion protein by cyclic amplification of protein misfolding. Nature.

[19] Concha-Marambio L (2016). Detection of prions in blood from patients with variant Creutzfeldt-Jakob disease. Sci. Transl. Med..

[20] Bougard D (2016). Detection of prions in the plasma of presymptomatic and symptomatic patients with variant Creutzfeldt-Jakob disease. Sci. Transl. Med..

[21] Moda F (2014). Prions in the Urine of Patients with Variant Creutzfeldt–Jakob Disease. N. Engl. J. Med..

[22] Morales R, Duran-Aniotz C, Diaz-Espinoza R, Camacho MV, Soto C (2012). Protein misfolding cyclic amplification of infectious prions. Nat. Protoc..

[23] Saá P, Castilla J, Soto C (2006). Ultra-efficient Replication of Infectious Prions by Automated Protein Misfolding Cyclic Amplification. J. Biol. Chem..

[24] Chen B, Morales R, Barria MA, Soto C (2010). Estimating prion concentration in fluids and tissues by quantitative PMCA. Nat. Methods.

[25] Mays CE (2011). *In vitro* amplification of misfolded prion protein using lysate of cultured cells. PLoS One.

[26] Bonda DJ (2016). Human prion diseases: surgical lessons learned from iatrogenic prion transmission. Neurosurg. Focus.

[27] Orrù CD (2017). RT-QuIC Assays for Prion Disease Detection and Diagnostics. in. Methods in molecular biology (Clifton, N.J.).

[28] Lacroux C (2014). Preclinical detection of variant CJD and BSE prions in blood. PLoS Pathog..

[29] Gill ON (2013). Prevalent abnormal prion protein in human appendixes after bovine spongiform encephalopathy epizootic: large scale survey. BMJ.

[30] Llewelyn C (2004). Possible transmission of variant Creutzfeldt-Jakob disease by blood transfusion. Lancet.

[31] Peden AH, Head MW, Diane LR, Jeanne EB, James WI (2004). Preclinical vCJD after blood transfusion in a PRNP codon 129 heterozygous patient. Lancet.

[32] Wroe SJ (2006). Clinical presentation and pre-mortem diagnosis of variant Creutzfeldt-Jakob disease associated with blood transfusion: a case report. Lancet.

[33] Douet JY (2017). Distribution and Quantitative Estimates of Variant Creutzfeldt-Jakob Disease Prions in Tissues of Clinical and Asymptomatic Patients. Emerg. Infect. Dis..

[34] Chen C, Dong X-P (2016). Epidemiological characteristics of human prion diseases. Infect. Dis. poverty.

[35] Walker LC, Jucker M (2015). Neurodegenerative diseases: expanding the prion concept. Annu. Rev. Neurosci..

[36] Soto C, Pritzkow S (2018). Protein misfolding, aggregation, and conformational strains in neurodegenerative diseases. Nat. Neurosci..

[37] Salvadores N, Shahnawaz M, Scarpini E, Tagliavini F, Soto C (2014). Detection of Misfolded Abeta Oligomers for Sensitive Biochemical Diagnosis of Alzheimer’s Disease. Cell Rep..

[38] Shahnawaz M (2017). Development of a Biochemical Diagnosis of Parkinson Disease by Detection of alpha-Synuclein Misfolded Aggregates in Cerebrospinal Fluid. JAMA Neurol..

[39] Saijo E (2017). Ultrasensitive and selective detection of 3-repeat tau seeding activity in Pick disease brain and cerebrospinal fluid. Acta Neuropathol..

[40] Groveman BR (2018). Rapid and ultra-sensitive quantitation of disease-associated alpha-synuclein seeds in brain and cerebrospinal fluid by alphaSyn RT-QuIC. Acta Neuropathol. Commun..

[41] Fairfoul G (2016). Alpha-synuclein RT-QuIC in the CSF of patients with alpha-synucleinopathies. Ann. Clin. Transl. Neurol..

